# Negotiating markets for health: an exploration of physicians’ engagement in dual practice in three African capital cities

**DOI:** 10.1093/heapol/czt071

**Published:** 2013-09-26

**Authors:** Giuliano Russo, Barbara McPake, Inês Fronteira, Paulo Ferrinho

**Affiliations:** ^1^International Public Health and Biostatistics Unit, Instituto de Higiene e Medicina Tropical, Universidade Nova de Lisboa and Centre for Malaria and Tropical Diseases, Associated Laboratory, Lisbon, Portugal and ^2^Institute for International Health Development, Queen Margaret University, Edinburgh, UK

**Keywords:** Dual practice, multiple job-holding, human resources for health, physicians in Africa, Cape Verde, Guinea Bissau, Mozambique, health system research in low-income countries

## Abstract

Scarce evidence exists on the features, determinants and implications of physicians’ dual practice, especially in resource-poor settings. This study considered dual practice patterns in three African cities and the respective markets for physician services, with the objective of understanding the influence of local determinants on the practice. Forty-eight semi-structured qualitative interviews were conducted in the three cities to understand features of the practice and the respective markets. A survey was carried out in a sample of 331 physicians to explore their characteristics and decisions to work in public and private sectors. Descriptive analysis and inferential statistics were employed to explore differences in physicians’ engagement in dual practice across the three locations. Different forms of dual practice were found to exist in the three cities, with public physicians engaging in private practice outside but also inside public facilities, in regulated as well as unregulated ways. Thirty-four per cent of the respondents indicated that they worked in public practice only, and 11% that they engaged exclusively in private practice. The remaining 55% indicated that they engaged in some form of dual practice, 31% ‘outside’ public facilities, 8% ‘inside’ and 16% both ‘outside’ and ‘inside’. Local health system governance and the structure of the markets for physician services were linked to the forms of dual practice found in each location, and to their prevalence. Our analysis suggests that physicians’ decisions to engage in dual practice are influenced by supply and demand factors, but also by how clearly separated public and private markets are. Where it is possible to provide little-regulated services within public infrastructure, less incentive seems to exist to engage in the formal private sector, with equity and efficiency implications for service provision. The study shows the value of analysing health markets to understand physicians’ engagement in professional activities, and contributes to an evidence base for its regulation.

**KEY MESSAGES**

- Different forms of dual practice were found to exist in three African capital cities, with public physicians engaging in private practice outside but also inside public facilities, in regulated as well as unregulated ways.
- Thirty-four per cent of physicians indicated they engaged in public practice only. Eleven per cent indicated that they engaged in private practice only. The remaining 55% indicated that they engaged in some form of dual practice. The proportion of dual practitioners was found to be consistent across the three locations.
- Local health system governance and structure of the markets for physician services were linked to the forms of dual practice found in each location, and to their prevalence.
- Analysing structure and features of local markets for physician services is key to understand physicians’ decisions to engage in dual practice, and identify suitable policy options.

## Introduction

Physicians’ simultaneous engagement in multiple clinical professions is very common worldwide. Those who combine a mix of public and private sector work are most often referred to as ‘engaging in dual practice’ (Eggleston and Bir 2006; García-Prado and González 2011; Socha and Bech 2011), as ‘multiple or dual job-holders’ (Berman and Cuizon 2004; Jan *et al.* 2005), or as undertaking ‘multiple income-generating activities’ (Jumpa *et al.* 2007). The term ‘moonlighting’ (Biglaiser and Albert Ma 2007) has also been used, implying an element of illegality about the private sector work of public sector physicians.

Several studies have documented the existence as well as prevalence of dual practice in low, middle and high-income countries alike. Gruen *et al.* (2002) refer to a study in Bangladesh according to which up to 80% of public sector doctors engage in some form of dual practice. Berman and Cuizon (2004) report a similar proportion among general practitioners in Indonesia, and in Egypt. In Thailand, Prakongsai *et al.* (2003) estimated that 69% of public sector physicians had private sector activities. In Europe, Humphrey and Russell (2004) find that in the UK, 63% of public hospital consultants and specialists maintain a private practice alongside their job in the National Health Service, while Dolado and Felgueroso (2007) found that 20% of Spanish public sector physicians have a second job.

There is consensus in the literature that not enough evidence exists on the net impact of the practice on population access to services (Socha and Bech 2011). Berman and Cuizon (2004) argue that dual practice may reduce quantity and quality of public services, as it undermines the ethos of public service, creates grounds for absenteeism and shirking and diverts resources and patients to private practice. A common argument in favour of dual practice is that it helps retain skilled physicians in low-paid public posts (Ferrinho *et al.* 2004; Jan *et al.* 2005), and creates an opportunity for physicians to update their skills in the private sector. González (2004) argues that dual practice might improve service quality in the public sector, since physicians would seek to establish their private sector credentials through quality services offered in the public sector. The possibility of practising legally both in public and private clinics has also been credited with reducing unofficial payments in the former (García-Prado and González 2007). Eggleston and Bir (2006) suggest that, through price discrimination, dual practice allows more services to be offered to the public, and at a more equitable price.

The opportunity to generate additional income has largely been considered as physicians’ main motivation to engage in dual practice (Ferrinho *et al.* 1998), although improving professional prestige (González 2004) and complementarities between public and private practices (Chawla 1996) have also been cited as additional factors. Ferrinho *et al.* (2004) conclude that, particularly in low-income countries, health professionals engage in dual practice because of the gap existing between professional and economic expectations and what public employment can offer.

Despite the literature on the subject, evidence is scarce on features and determinants of dual practice, particularly in resource-poor settings. This article seeks to explain differences in the patterns of dual practice in three African cities by analysing physicians’ professional engagement and the characteristics of health governance and local markets for medical services.

## Background

The three locations were selected not only because of their similarities in terms of cultural heritage and organization of the respective health systems, but also because of their different stages of economic development, which was expected to offer insight into the impact of evolving health markets on physician dual practice.

Granted independence from Portugal in 1975, Cape Verde has made the transition to middle-income status. In 2010 Cape Verde’s health expenditures were USD154 per capita, the majority of which was public (Table 1). The Agostinho Neto Central Hospital (ANCH) in Praia is considered the pinnacle of curative services across the ten islands; the medical private sector is liberalized, and private practice within public premises is legally permitted.

**Table 1:** Selected characteristics for the three study locations

Characteristic	Cape Verde	Guinea Bissau	Mozambique
Country’s GDP per capita (PPP)^a^	3984	1186	942
Total health expenditures per capita (current prices, 2010 USD)	154.6	46.9	21.3
Private health expenditures per capita (current prices, 2010 USD)	38.6	42.2	6.0
Position in the Human Development Index (out of 187)^b^	133	176	184
Physicians registered in the country^c^	400	172	1105
Physicians residing in the capital cities^c^	131	127	487
Population in capital cities^d^	131 453	387 908	1 178 116

Note: Gross Domestic Product (GDP); USD Purchasing Power Parity (PPP); United Nations Development Program (UNDP).

^a^The World Bank (2012).

^b^UNDP (2011).

^c^National Medical Councils (2012).

^d^National Statistical Institutes.

Marred by civil war and political instability since independence, Guinea Bissau is one of Africa’s poorest and least developed countries. Health expenditures are USD47 per capita. The public health sector network consists of seven hospitals, including Bissau’s Simão Mendes Central Hospital (SMCH). Private health expenditure represented 90% of total health expenditure in 2010 (World Bank 2013). Private health practice was legalized in 1986, but no regulation exists of private health practice within public institutions. ‘Private’ services (those for which a fee is charged) are available across all public facilities (Einardóttir 2011).

After emerging from civil war in 1992 with its health system in tatters, Mozambique is currently developing rapidly because of the recent discovery of mineral resources, which has increased national income as well as inequalities (MPD 2010). There were 1105 physicians registered in the country, most of who trained in the country’s four medical schools. The Maputo Central Hospital (MCH) is Mozambique’s largest treatment institution, based in the country’s densely populated capital city; private provision of health services was legalized in 1992, including private services offered through the MCH ‘special clinic’ (GoM 1992).

## Methodology

**Figure 1:**
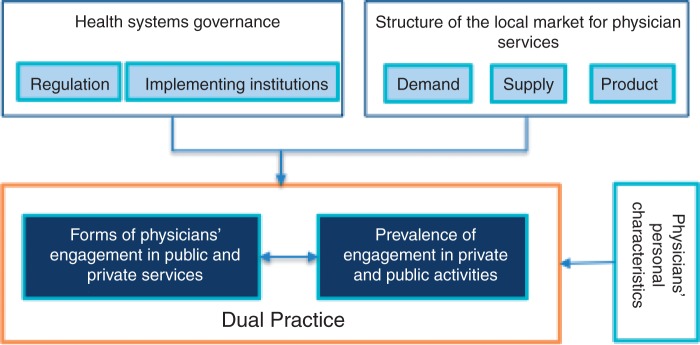
Conceptual framework for the analysis of physician dual practice.

The present study set out to investigate physician dual practice in three African capital cities, with the objective of understanding the influence of local conditions on its forms and prevalence. According to our framework (Figure 1), the form and prevalence of physicians’ simultaneous engagement in public and private clinical activities critically depend on local factors linked to the health system’s governance (regulation and implementing institutions), as well as to the structure of the market for physician services (supply, demand and product). Physicians’ personal characteristics are considered independent of these local determinants and are not explored in this article.

We define the product as all clinical services provided by physicians in the public and private sectors. Given the lack of data to apply alternatives, we used the simple geopolitical boundary approach (Zwanzinger *et al.* 1994) to identify the geographical scope of the market. We discuss the limitations of these two methodological choices in our discussion.

A two-phase, sequential mixed methods study was employed to understand physicians’ engagement in dual practice and the respective local markets (Creswell 2002). The first phase was a qualitative exploration of issues identified as relevant by the economic and industrial organization literature, and in a second data collection phase, surveys were conducted among physicians to take stock of dual practice in the three capital cities and measure quantitatively concepts identified as important through the interviews. Multiple secondary data sources were used to gain further perspectives on the market for physician services in the three cities. The Ministry of Health’s (MoH) activity and personnel statistics were used to analyse demand and supply of services in the three capital cities; regulatory and salary information were retrieved from parliamentary bills and ministerial decrees.

### Qualitative interviews

We individually interviewed a purposive sample of 48 public and private sector physicians, as well as key health policy-makers identified with the help of local MoHs and Medical Councils (Table S1 in supplementary data). The interview guide drew on the dual practice as well as the health systems and industrial organization literature, and aimed to explore issues identified as relevant including: the features of demand and supply of physicians’ services, characteristics of the product offered, market structure and regulation, governance and incentives (see interview guide in the supplementary data).

Interviews were all conducted by the principal investigator and by one research assistant, tape-recorded and transcribed. The directed content analysis approach (Hsieh and Shannon 2005) was employed to analyse the interviews, with coding categories derived from the relevant dual practice literature. Triangulation across each group of respondents and relevant documentary evidence were used to gain a deeper understanding of contrasting opinions on key themes.

### The surveys

Surveys were used to collect data from a representative sample of physicians working in the three capital cities on physician characteristics, their working time allocation, motivation to work in public and private sectors and perceptions of regulation. MoHs’ and Medical Councils’ physician databases were used to identify as complete as possible a list of registered physicians in the three cities (Table S2 in the statistical supplementary data).

Given the limited size of the health sector in Praia and Bissau, the survey questionnaire was administered in person to 100% of medical doctors working in these two capital cities. The response rates for Praia and Bissau were 79.7% and 80.5%, respectively. For Maputo, where the physician population was considerably larger, we surveyed a simple random sample of physicians drawn from the MoH and Medical Council databases. As many physicians from the original sample turned out to be retired, dead or working outside Maputo upon contact, we proceeded to identify a further sampling fraction. As a sample of 30% of the population is, under certain conditions, considered acceptable for estimating prevalence of common characteristics (Aguiar 2007; Lohr 2009), we stopped recruiting survey participants when our target proportion was reached. Of the overall estimated population across the three cities 51.8% agreed to participate, 7.1% refused and 41.1% could not be located (Table S2 in the statistical supplementary data).

The survey questionnaire contained 31 questions about physicians’ general characteristics, allocation of time through professional activities, public and private sector work characteristics and regulation of the profession (see survey questionnaire in the supplementary data). Some questions were designed to produce either multiple choice or closed-ended answers, while for others, respondents were asked to state their preferences in a 4-degree Likert scale. In order to measure the association between the dependent and independent variables and its statistical significance, adjusted odds ratio, confidence intervals (CIs) for the adjusted odds ratio, Wald statistics and respective *P*-values were computed.

The surveys were conducted in three separate teams by 10 local data collectors with a health sector background, trained and supported by a team of researchers from the Institute of Hygiene and Tropical Medicine (IHMT). The study had the approval of the IHMT ethics committee. For the fieldwork in Mozambique a further approval was requested and obtained from the Mozambican MoH’s bio-ethics committee. IHMT approval was considered sufficient by the MoHs in Cape Verde and Guinea Bissau. Informed consent was obtained from all participants in the interview and survey phases.

## Results

In this section, survey and interview results are described to explore the shape and prevalence of physicians’ engagement in dual practice in the three cities, as well as the influence of physicians’ personal characteristics on this engagement. Evidence is presented from interviews and document analysis on health system governance and the structure of markets for physicians’ services.

### Forms of dual practice

Our qualitative and quantitative data identified different forms of dual practice in the three cities. A substantial proportion of public sector physicians reported engaging with four types of private clinical activities, involving different levels of public resource use, regulation, quality and prices:

- Private practice ‘outside’ public practice: medical practitioners operate or work in distinct private facilities, unconnected physically or institutionally to public sector facilities. This form of private services was found to be ‘thriving’ in Maputo, ‘developing, but still limited to outpatient services’ in Praia, and ‘underdeveloped and very diverse’ in Bissau.
- Private practice ‘beside’ public practice: medical services offered by physicians in specialized institutions through separate venues adjacent to the main hospital. In this study the MCH ‘special clinic’ is the sole example, although similar arrangements are described in the literature in other countries (Shirom 2001; Suwandono *et al.* 2001). These services are officially regulated and use separate resources and accounting systems; patients pay higher prices than in the main public facility. In the case of MCH, a monthly fixed payment is made by the ‘special clinic’ to the main hospital for ‘general expenditures’ (McPake *et al.* 2011).
- Private practice ‘within’ public practice facilities: dedicated medical services offered within public premises outside public sector hours, using public resources, partly regulated by the hospital or by the department running them. This is known as ‘special services’ in MCH and also occurs in ANCH. The quality of such services does not appear to differ much from those offered publicly, and patients typically seek them to avoid waiting. Customers pay out-of-pocket a fee higher than that applicable in the main public facility, a fixed proportion of which is transferred to the hospital/department.
- Private practice ‘integrated’ into public practice: medical services provided by resident physicians in public facilities, held within public premises, using public resources, and typically offered with no time restrictions and during public sector shifts but on the basis of informal payment (very common in Bissau, especially in SMCH). Such services are illegal, and therefore erratic, not regulated and seldom openly reported. Patients pay a variable fee, typically higher than the official public sector fee, but lower than the private sector standard, and none of this is transferred back to the hospital.

In Praia, private services exist mostly ‘outside’ public ones, with the exception of the disappearing ‘*consultas complementares*’, in the ‘within’ public facilities category; in Maputo private services would appear ‘outside’ but also ‘beside’ and ‘within’ the public sector through its MCH ‘special clinic’ and ‘special services’; in Bissau ‘outside’ private services exist, but private practice is mostly integrated into public premises through informal charges. According to the interviews illegal payments are pervasive in the Guinean healthcare system, and serious doubts exist that patients can access any physician service at all without having to pay a string of arbitrary and unpredictable fees.

… ok, this is what happens in the public [sector]: a patient arrives, the outpatient visit [fee] is charged, which is 1,000 West African Franc (Xof) and this amount is for the hospital. Then I am seeing the patient, and I ask for some [payment] for myself. Perhaps I can ask around 2,000XOF, and these 2,000 are all for me. Then the nurse who attends the patient with me also asks for some payment, and so does the attendant. It is a whole set of undue payments*.* [Senior SMCH specialist]

### Prevalence of physicians’ engagement in dual practice

Of the 331 physicians surveyed, two were excluded because they were not in clinical practice. 54.1% were male, with a mean age of 44.8 years (Standard Deviation (SD) = 9.5). In total, 73.1% were married and 90.9% had dependents: 4.1 on average (SD 3.5). And, 46.6% had a second physician in the family, 65.2% had a specialty, and 11.4 (SD 8.3) years was the mean years of professional practice; 17% practiced outside the capital city. In comparison with the other two cities, Bissau’s physicians were more male (71.9%), had more dependents—7.7 (SD 4.0) and a larger proportion of them had a specialization (see Table S3 in the statistical supplementary data).

Across all three cities, 34% of those surveyed indicated that they practised in the public sector only and this response was similar in each city. 11% indicated that they practised in the private sector only (highest in Bissau: 16% and lowest in Maputo: 6%). The remaining 55% engaged in some form of dual practice (54% in Praia, 57% in Maputo and 53% in Bissau) (Table 2).

**Table 2:** Physicians engagement in dual practice in the three locations

Type of physician professional engagement	Praia		Bissau		Maputo		Total	
	% [95% CI]	*N*	% [95% CI]	*N*	% [95% CI]	*N*	% [95% CI]	*N*
Public only	33.0%	36	31.6%	30	36.8%	46	34.0%	112
	[28.9–37.1]		[27.2–35.9]		[33.0–40.6]		[31.7–36.4]	
Total dual practice	54.1%	59	52.6%	50	56.8%	71	54.7%	180
	[44.7–63.5]		[42.6–62.7]		[48.1–65.5]		[49.3–60.1]	
Public and ‘private inside’ public facilities	2.7%	3	17.9%	17	5.6%	7	8.2%	27
	[1.3–6.8]		[13.5–22.8]		[1.8–9.4]		[5.9–10.6]	
Public and ‘private outside’ public facilities	42.2%	46	23.2%	22	26.4%	33	30.7%	101
	[38.1–46.3]		[18.8–27.5]		[22.6–30.2]		[28.3–33.0]	
Public and ‘private outside and inside’ public facilities	9.1%	10	11.58%	11	24.8%	31	15.8%	52
	[5.1–13.2]		[7.2–15.9]		[21.0–28.6]		[13.46–18.16]	
Private only	12.84%	14	15.8%	15	6.4%	8	11.2%	37
	[8.7–16.9]		[11.4–20.2]		[2.6–10.2]		[8.9–13.6]	
Total	100.0%	109	100.0%	95	100.0%	125	100.0%	329

*Source:* Dual practice survey (2012).

The survey did not allow for the separation of the three categories of ‘inside’ dual practice elaborated above. ‘Outside’ dual practice was indicated by 30% of physicians and ‘inside’ dual practice by 8% of respondents (categories b, c and d above), while 16% declared engaging in both ‘outside’ and ‘inside’ forms simultaneously (categories a–d). Praia recorded the highest proportion of public physicians indicating engaging in dual practice ‘outside’ the public sector (42.2%), Bissau the highest proportion who indicated that their dual practice consisted of a mix of public and ‘inside’ private practice only (17.9%), whereas Maputo showed the highest proportion of physicians engaging in both forms of dual practice simultaneously (24.8%).

There are statistically significant differences in the simultaneous engagement in the formal public and private sectors between Bissau and the other two cities, as well as for public sector exclusive employment between Bissau and Praia. The differences in the percentages of physicians engaging in all forms of dual practice in the three cities were statistically significant (*χ*^2 ^= 12.1; *P* < 0.01).

### Health systems’ governance

Regulation and implementing institutions were described in the interviews as ‘relatively strong’ in Cape Verde, ‘hopelessly weak’ in Guinea Bissau and ‘of mixed strength’ in Mozambique (Table 3). The legal documents we reviewed showed that in Cape Verde private practice is liberalized (GoCV 1991, 1989), and physicians’ private practice within public premises legally permitted outside regular shifts (GoCV 1987). Public sector physicians’ working time is limited to between 8.30am and 3.30pm, with the explicit objective of allowing health personnel to undertake private work in the afternoon.

**Table 3:** Selected health governance and market structure characteristics

Health system’s governance and market characteristics	Praia	Bissau	Maputo
Regulation and regulatory institutions	Developed and formal regulation in place for private sector and ‘special services’. Comparatively strong implementing institutions, from MoH to Hospital management and Medical Council	Absent. Week implementing and governance institutions. At the time of the fieldwork, the SMCH was under administration, and the Medical Council did not have a list of physicians working in Bissau	Patchy regulation, only selectively applied. Some institutions (General government and MCH) stronger than others (Medical Council). Government attempts to ban Special Services were flatly ignored by MCH
Physicians’ opinions about dual practice (DP) regulation	DP should be regulated (89.1%), by the Government (33.3%), by the MoH (70.7%) and by the Medical Council (73.7%)	DP should be regulated (95.8%), by the Government (50.0%), by the MoH (60.4%) and by the Medical Council (78.1%)	DP should be regulated (71.8%), by the Government (12.9%), by the MoH (37.1%) and by the Medical Council (53.2%)
Formal private practice outside public facilities	Developed and regulated, although mostly limited to outpatient visits	Very limited, predominantly low-cost and scarcely regulated	Thriving and high-cost, patchily regulated
Private practice inside public facilities	‘*Consultas complementares*’ legalized and regulated within ANCH (private practice ‘within’). It is however limited in size, and mostly related to surgical operations and equipment-intensive tests	Unregulated, it is however very common and ‘integrated’ across all the public sector. Informal illegal charges reported to be ubiquitous	Existing as both little-regulated ‘Special Services’ within hospital departments, and more formalized ‘special clinic’ services ‘beside’ MCH public services.
Proportion of specialists among physicians surveyed	Moderate proportion of specialists (65.1%)	Highest number of specialists (75.6%)	Lowest proportion of specialists (56.0%) across the three locations surveyed, possibly linked to existence of local training capacity for basic medical degrees
Physician density in capital city	Highest physicians density of the three locations, comparable with middle- and high-income countries (9.96/10 000)	Lowest physicians density (3.27/10 000)	Average physicians density (6.64/10 000)
Public sector pay	Comparatively high and decompressed (USD903–1802)	Low and compressed (USD315–344)	Low, but decompressed (USD645–989)
Private service prices	Moderate (USD29.22 for outpatient visit)	Comparatively low (USD5.97 for outpatient visit)	High (USD34.99 for outpatient visit)
Demand for public medical services	Moderate, with some waiting list for the central hospital	Moderate, possibly because of burdensome illegal charges	High, although legal moderating fees limiting access to tertiary-care hospitals

In Guinea Bissau private medical regulation is very limited, consisting of a 1986 law legalizing private practice (GoGB 1988) and a 1992 decree establishing the requirements to start a private clinic. Private practice in hospital facilities is neither recognized or regulated, nor are hospital fees and physician working hours (GoGB 1992). However, evidence exists that illegal charges are ubiquitous (Einardóttir 2011).

Interviews and documents revealed that regulation in Mozambique is patchy, as most private and public service standards are defined, but exceptions are specified for many hospitals, especially for the MCH. The MCH ‘special clinic’ is legally permitted (GoM 1992), but other forms of special services within public facilities are not. In 2007 the MoH formally banned special services from public facilities (MoH 2007). The ban was enforced throughout the country, but successfully ignored by the MCH.

Regulatory institutions were reported to be relatively consolidated in Cape Verde. The MoH appears to have substantial influence on the ANCH’s management, and the national Medical Council is a consolidated and credible institution, holding regular elections and influencing health policy. At the other end of the spectrum, institutions in Guinea Bissau are weak and discredited, with a cash-strapped and ineffectual MoH, a leaderless central hospital and a nascent Medical Council. Mozambique has strong central institutions linked to the party system, a rather independent MCH, and a Medical Council still in the process of consolidation. The MCH physician lobby was reported to substantially influence national health policy, often in an antagonistic relationship with the MoH, although senior MoH appointments are typically drawn from the central hospital. MCH’s ability to ignore the ministerial ban of hospital special services illustrates their power and influence within the sector.

I cannot go around and check whether my co-workers are at work here at the department and ask: ‘where were you?’. This is the independent republic of the Maputo Central Hospital! [Senior MCH specialist]

When asked whether dual practice should be regulated, the vast majority of physicians responded positively (84.5%), with Maputo physicians showing the least enthusiasm for regulation (71.8%) (Table 3). Although regulation may be seen positively as ‘bringing clarity about the rules of the game for all physicians’ [junior physicians in Maputo], many complain that the current regulation does not do enough to foster private practice outside:

… they say ‘go and open your own private clinic’, but then you have to pay hefty taxes, and loans to start your business carry regular bank interest rates. [Senior public sector physician from Bissau]

### The structure of the market for medical services

From the supply-side perspective, Praia is the capital city with the largest number of physicians per capita (10.5 per 10 000 inhabitants), followed by Maputo and Bissau. The overall proportion of specialists averages 65.2% among the physician population in the three cities, with Bissau being the market with the largest proportion of specialists (Table 3).

In comparison with the other two countries, Cape Verde’s doctors are paid a comparatively higher salary within a decompressed salary structure (ranging from USD903 to 1802 monthly at current exchange rates) and receive pension benefits. Remunerative exclusivity contracts are available for those physicians in key political posts who agree to forego private practice. The possibility exists for junior doctors to complement their salary through emergency shifts and rural posting allowances. In Guinea Bissau, salaries are comparatively low and compressed (between USD315 and 344), and there are limited allowances, negligible pension schemes and little government support for the private sector in the country’s insecure business environment. In Mozambique an intermediate situation was found, with comparatively higher but still relatively compressed public sector salaries (USD645–989), and no exclusivity contracts, although revisions to physicians’ public sector contracts are currently under consideration. Almost all dual practitioners (98.7%) reported that ‘increasing one’s income’ was an important or very important reason for engaging in private sector activities. Dissatisfaction with public sector salaries is widely seen as creating the necessity for physicians to engage in dual practice:

If I were paid enough [by the government] don’t you think I would gladly give up this life of hopping from one private practice to the other? This is not life! [Senior MCH physicians from Maputo]

Praia was the capital city with the largest number of private facilities per capita (2.21 per 10 000 inhabitants), although Maputo was the one with the largest absolute number (142 for its 1 178 116 inhabitants). By some distance, Bissau appeared to have the least developed private health sector (0.7 facilities per 10 000 people), with very little inpatient care and surgical capacity to speak of, although faith-based Non-Governmental Organizations (NGOs) were reported to play a significant part in the supply of higher quality physician services.

Public sector fees are mostly low, when illegal charges are not applied. From our survey, reported private sector prices appear comparatively low in Bissau (USD6 for an outpatient visit at market exchange prices), intermediate in Praia (USD29 for outpatient visits) and high in Maputo (USD35) (Table 3).

On the demand side, activity statistics and interviews with key informants suggested that utilization of public sector physician services is the highest in Maputo. Rationing mechanisms are in place in all three cities acting to moderate population access to such services, through referral systems (Praia and Maputo); legal payments (Maputo); and illicit charges (Bissau). In Praia’s ANCH, waiting lists of less than 30 days exist for most specialties. In Bissau, specialist visits are available for some specialties at the SMCH, no organized waiting list is in place, and specialists reported being able to see all their patients in their shift, although it is unlikely patients can access such services without paying illicit out-of-pocket fees (Einardóttir 2011). In Maputo, patients can access specialists’ visits in central and general public hospitals if referred from a lower institution, or upon out-of-pocket payment through ‘special services’ in MCH; illicit charges were reported to be uncommon.

Physicians’ own perceptions suggest that the demand conditions they face for private sector services are more favourable in Maputo than in Praia and Bissau, explained by Mozambique’s booming economy, its capital city’s growing middle class and its population’s consequent higher ability to pay. Interviews with physicians described a ‘saturated’ private sector market in Praia, a ‘difficult’ market in Bissau (limited demand and uncertainty around recovering initial investment) and an ‘aggressive’ one in Maputo (increasing competition among physicians). The proportion of private sector physicians believing that lack of demand is a constraint to doing further private sector work is considerably smaller in Maputo (14.5%) than in Praia (45.6%) and Bissau (48.8%) (*P* <0.01).

## Discussion

The present study adopted qualitative as well as quantitative methods for understanding dual practice and the market for physician services in three African cities, which is a somewhat heterodox method for analysing markets. This was primarily justified by the absence of appropriate quantitative data sets to undertake more orthodox forms of analysis, but also by the innovative contributions that recent studies have shown qualitative information from market actors can offer to this kind of investigation (Ranson *et al.* 2012; Russo and McPake 2010).

We made simplifying choices in defining the markets in terms of geographical and product boundaries. Our identification of the market within the geographical boundaries of the three cities had limitations: the flow of patients from and to the three capitals’ healthcare networks is considerable. Our analysis also considered the broad market for medical services, regardless of the type of service provided. Although recognizing that such generalizations may hide key features of particularly concentrated markets (Chang *et al.* 2011), we also observed that distinctions between types of services become blurred in the field, and as suggested in the literature, market boundaries in less sophisticated health systems turn out to be very porous (García-Prado and González 2011). We achieved good response rates to the survey. Although almost 50% of those sampled in Maputo were not available, less than 8% declined to respond. The remainder proved not to qualify for inclusion although the register from which we drew the sample implied they should. This does not then create a significant problem of bias in the measured responses.

Given the existence of several ways of mixing public and private services, more than one form of dual practice may exist in poorly regulated settings, and future work on the subject will need to take this into account. It is helpful to link the phenomenon of ‘outside’ dual practice with variants of public–private mix increasingly more integrated with public provision, as it is likely that both users and physicians substitute between these options. Our survey results appear to show that fewer physicians engage in simultaneous public and private sector activities (55%) than has been estimated by previous studies among public physicians (Gruen *et al.* 2002; Prakongsai *et al.* 2003) although Gupta and Dal Poz 2009 show a much smaller engagement in dual practice for physicians in Mozambique. The studies above obviously refer to very different geographical settings; however, if we consider as dual practitioners also those public sector physicians who offer private services inside public facilities, then the overall prevalence of dual practice would be broadly consistent across the three locations, as well as with the existing literature. It is also unlikely that the full extent of ‘integrated’ dual practice was captured by the survey as physicians may be least likely to admit to this form of dual practice, or perhaps to recognize it as dual practice, if willing to admit to it.

Our findings show that, consistent with the literature from physicians’ perspectives, low public pay creates the necessity to resort to private service provision (Ferrinho *et al.* 1998; Meng *et al.* 2009). However, the three case studies suggest that governance may be playing a significant role in shaping patterns of dual practice; when governance is strong, as in Praia, regulation may protect public characteristics of services within time and space boundaries and may force private sector activity ‘outside’ in either time or space, to where it is formally recognized as dual practice. Conversely, where governance is weak, as in Bissau, physicians seem to prefer to offer private services within public facilities where it may escape regulation altogether. This preference may also be shaped by the lower ability to pay in the Bissau market than the Praia market: potential profitability may not support the maintenance of separate, external facilities. Interestingly, physicians appear to welcome the regulation-driven separation represented by the ‘outside’, ‘beside’, ‘within’ and ‘integrated’ categories. One explanation of this is that it might force customers to self-select across differently priced services, thus enabling physicians to extract consumers’ surplus more efficiently (Hirshleifer 1976).

The existence of ‘integrated’ private services within the public sector may jeopardize accessibility of services, as patients may be required at any stage to pay out-of-pocket for services they are entitled to receive free of charge. Under this premise, regulated ‘special services’ ‘within’ or ‘beside’ public facilities may represent an effective way to protect public services, by reducing or removing informal charges. As some authors point out, when these are brought to light and clearly defined, as in Praia’s ANCH’s ‘*consultas complementares*’, they might be welfare-enhancing, as they might provide wider product choice to consumers, benefiting physicians and patients alike. However, when these ‘special services’ are loosely regulated, as within the MCH’s departments, they may instead generate confusion and reduce the space for public services (McPake *et al.* 2011). In a theoretical model of what we are categorizing as the ‘within’ model, McPake *et al.* 2007 show that, in the organization of a hospital’s public and ‘on-payment’ services, equity enhancing and equity reducing outcomes can result, depending on whether hospitals maximize profit or revenue, and on the inter-relationships between demand functions for the different services. A similar analysis could be applied to the individual physician’s decision to allocate effort across two services (public and private provision) and may also identify equity enhancing and equity reducing scenarios.

Government attempts to regulate dual practice in low-income contexts will have to take this added complexity into consideration, which is consistent with some of the existing literature on this subject (for example González and Macho-Stadler 2013).

## Conclusions

The present study looked at physicians’ engagement in dual practice activities in three African capital cities through a mix of quantitative and qualitative methods, with the objective of increasing understanding of the influence of local market and governance characteristics on physicians’ decisions. We conclude that several forms of dual practice seem to exist in low-income settings, and the blurred definition of the boundaries between public and private market may play a critical role in understanding physicians’ decisions to engage in multiple activities. As this is likely to have repercussions for the effectiveness of government policies to influence dual practice, further analysis of local markets for health in scarcely regulated settings is needed to support design of more effective policies to improve access to physicians’ services.

## Supplementary data

Supplementary data are available at *Health Policy and Planning* online.

## Funding

The study received funding from the Calouste Gulbenkian Foundation, Portugal (grant No. FCG_120359) and from the World Health Organisation's Department of Human Resources for Health (grant No. HQHRH1002945).

## Conflict of interest

None declared.

## Supplementary Material

Supplementary Data

Translated Abstracts

## References

[czt071-B1] Aguiar P (2007). Guia prático Climepsi de Estatística em Investigação Epidemiológica.

[czt071-B2] Berman P, Cuizon D (2004). Multiple public-private jobholding of health care providers in developing countries. An exploration of theory and evidence. Issue paper - private sector.

[czt071-B3] Biglaiser G, Albert Ma C (2007). Moonlighting: public service and private practice. The RAND Journal of Economics.

[czt071-B4] Chang DC, Shiozawa A, Nguyen LL, Chrouser KL, Perler BA, Freischlag JA, Colombani P, Abdullah F (2011). Cost of inpatient care and its association with hospital competition. Journal of the American College of Surgeons.

[czt071-B5] Chawla M (1996). Public-private interactions in the health sector in developing countries: sharing of labor resources.

[czt071-B6] Creswell JW (2002). Research Design: Qualitative, Quantitative, and Mixed Methods Approaches.

[czt071-B7] Dolado JJ, Felgueroso F (2007). Occupational mismatch and moonlighting of Spanish physicians: Do couples matter?.

[czt071-B8] Eggleston K, Bir A (2006). Physician dual practice. Health Policy.

[czt071-B9] Einardóttir J (2011). Avaliação da Iniciativa de Bamako na Guiné-Bissau: “Sem dinheiro estás morto.” República da Guiné-Bissau, Ministério da Saúde, Evaluation Commissioned by the PNDS Implementation Unit and Sponsored by The World Bank.

[czt071-B10] Ferrinho P, Lerberghe WV, Fronteira I, Hipólito F, Biscaia A (2004). Dual practice in the health sector: review of the evidence. Human Resources for Health.

[czt071-B11] Ferrinho P, Lerberghe WV, Julien MR, Fresta E, Gomes A, Dias F, Gonçalves A, Bäckström B (1998). How and why public sector doctors engage in private practice in Portuguese-speaking African countries. Health Policy and Planning.

[czt071-B12] García-Prado A, González P (2007). Policy and regulatory responses to dual practice in the health sector. Health Policy.

[czt071-B13] García-Prado A, González P (2011). Whom do physicians work for? An analysis of dual practice in the health sector. Journal of Health Politics, Policy and Law.

[czt071-B14] Government of Cape Verde (GoCV) (1987). Decreto n.^o^ 45/87 de 16 de Maio.

[czt071-B15] GoCV (1989). Lei de base da Saúde.

[czt071-B16] GoCV (1991). Dec-Lei n.^o^ 183/91 de 28 de Dezembro.

[czt071-B17] Government of Guinea Bissau (GoGB) (1988). Decreto n.^o^ 29/88.

[czt071-B18] GoGB (1992). Despacho n.^o^ 20/GPM/92.

[czt071-B19] Government of Mozambique (GoM) (1992). Regulamento de prestação de cuidados de saúde por entidades privadas.

[czt071-B20] González P (2004). Should physicians’ dual practice be limited? An incentive approach. Health Economics.

[czt071-B21] González P, Macho-Stadler I (2013). A theoretical approach to dual practice regulations in the health sector. Journal of Health Economics.

[czt071-B22] Gruen R, Anwar R, Begum T, Killingsworth JR, Normand C (2002). Dual job holding practitioners in Bangladesh: an exploration. Social Science & Medicine.

[czt071-B23] Gupta N, Dal Poz MR (2009). Assessment of human resources for health using cross-national comparison of facility surveys in six countries. Human Resources for Health.

[czt071-B24] Hirshleifer J (1976). Price Theory and Applications.

[czt071-B25] Hsieh H-F, Shannon SE (2005). Three approaches to qualitative content analysis. Qualitative Health Research.

[czt071-B26] Humphrey C, Russell J (2004). Motivation and values of hospital consultants in south-east England who work in the national health service and do private practice. Social Science & Medcine.

[czt071-B27] Jan S, Bian Y, Jumpa M, Meng Q, Nyazema N, Prakongsai P, Mills A (2005). Dual job holding by public sector health professionals in highly resource-constrained settings: problem or solution?. Bulletin of the World Health Organization.

[czt071-B28] Jumpa M, Jan S, Mills A (2007). The role of regulation in influencing income-generating activities among public sector doctors in Peru. Human Resources for Health.

[czt071-B29] Lohr SL (2009). Sampling: Design and Analysis.

[czt071-B30] McPake B, Hanson K, Adam C (2007). Two-tier charging strategies in public hospitals: implications for intra-hospital resource allocation and equity of access to hospital services. Journal of Health Economics.

[czt071-B31] McPake B, Hongoro C, Russ G (2011). Two-tier charging in Maputo Central Hospital: costs, revenues and effects on equity of access to hospital services. BMC Health Services Research.

[czt071-B32] Meng Q, Jan S, Bian Y, Sun Q, Yu J (2009). Dual practice by public health providers in Shandong and Sichuan Provinces, China. Working Paper No. id:2225.

[czt071-B33] MoH (2007). Proibição de prestação de medicina privada em hospitais públicos.

[czt071-B34] Ministry of Planning and Development (MPD) (2010). Poverty and Wellbeing in Mozambique; the Third National Poverty Assessment.

[czt071-B35] Prakongsai P, Chindawatana W, Tantivess S, Mugen S, Tangcharoensathien V (2003). Dual practice among public medical doctors in Thailand. Report to the Health Economics and Financing Programme, The Health Economics and Financing Programme.

[czt071-B36] Ranson MK, Jayaswal R, Mills AJ (2012). Strategies for coping with the costs of inpatient care: a mixed methods study of urban and rural poor in Vadodara District, Gujarat, India. Health Policy and Planning.

[czt071-B37] Russo G, McPake B (2010). Medicine prices in urban Mozambique: a public health and economic study of pharmaceutical markets and price determinants in low-income settings. Health Policy and Planning.

[czt071-B38] Shirom A (2001). Private medical services in acute-care hospitals in Israel. The International Journal of Health Planning and Management.

[czt071-B39] Socha KZ, Bech M (2011). Physician dual practice: A review of literature. Health Policy.

[czt071-B40] Suwandono A, Gani A, Purwani S, Blas E, Brugha R (2001). Cost recovery beds in public hospitals in Indonesia. Health Policy and Planning.

[czt071-B41] The World Bank (2013). The World Bank Development Indicators database [online]. http://databank.worldbank.org/data/databases.aspx.

[czt071-B42] Zwanzinger J, Melnick G, Eyre K (1994). Hospitals and Antitrust: Defining Markets, Setting Standards. Journal of Health Politics, Policy and Law.

