# Supplementary material for: Negotiating markets for health: an exploration of physicians’ engagement in dual practice in three African capital cities
Source: Health Policy Plan. 2013 Sep 26;29(6):774–83. doi: 10.1093/heapol/czt071 (PMC4153303; doi:10.1093/heapol/czt071)
Supplement: Supplementary Data [file supp_29_6_774__index.html]

Negotiating markets for health: an exploration of physicians’ engagement in dual practice in three African capital cities — Supplementary Data 

# Negotiating markets for health: an exploration of physicians’ engagement in dual practice in three African capital cities

## Supplementary Data

files

**Files in this Data Supplement:**

- Supplementary Data - zip file
