# Supplementary figures and images for: Negotiating markets for health: an exploration of physicians’ engagement in dual practice in three African capital cities
Source: Health Policy Plan. 2013 Sep 26;29(6):774–83. doi: 10.1093/heapol/czt071 (PMC4153303; doi:10.1093/heapol/czt071)

Figure 1: conceptual framework for the analysis of physician dual practice


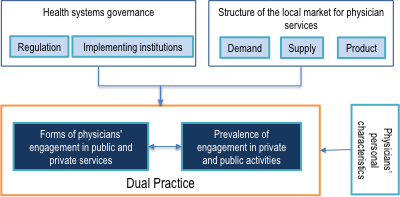

Supplement: Supplementary Data [file supp_czt071_suppl_data.zip › Figure 1_conceptual framework.docx]
