# Supplementary material for: Negotiating markets for health: an exploration of physicians’ engagement in dual practice in three African capital cities
Source: Health Policy Plan. 2013 Sep 26;29(6):774–83. doi: 10.1093/heapol/czt071 (PMC4153303; doi:10.1093/heapol/czt071)
Supplement: Translated Abstracts [file supp_czt071_czt071_Chinese.pdf]

目前，几乎没有证据研究医师双实践行为的特点、决定因素和此行为的含义。尤其针对资源贫乏的环境中，更少有证据涉及医师双实践行为。本文以 3 个非洲城市和各自的医师服务市场为例研究双实践模型，从而了解影响到双实践行为的当地因素。通过在 3 个城市进行的 48 个半结构式访谈，我们了解到 3 个城市各自的医师服务市场和实践行为的特点。本调查选取了 331 位医师作为样本，进而研究他们的个人特性和他们在公共卫生部门或私人卫生部门的工作决定。我们采用描述式分析和推断统计学来研究 3 个地域的医师对于双实践行为在不同程度上的投入。我们发现，在三个城市中存在不同形式的双实践行为。公共医师以监管方式和未监管方式同时从事外部私人的卫生工作和公共设施卫生工作。34%的被访者表示他们只在公共卫生部门工作，11%的被访者表示他们只在私人卫生部门工作。其余 55%的被访者表示他们存在双实践行为，其中 31%的医师处于公共卫生部门之外，8%的医师处于公共卫生部门之内，16%的人处于两者之间。当地医疗系统管理和医疗服务市场的结构与各地双实践行为的形式和普遍程度之间存在联系。经过分析我们发现，医师双实践行为的决策不仅受到供给和需求因素的影响，同时取决于公共医疗市场和私人医疗市场的差异区别程度。如果在公共医疗设施中提供低监管式服务，在考虑到效率和公平对提供服务的结果的时候，医师们失去了在正式私人部门中工作的动机。本研究说明分析医疗市场对于了解医师从事专业活动的价值。同时，本研究内容对于医疗市场的监管提供了事实依据。
