# Supplementary material for: Negotiating markets for health: an exploration of physicians’ engagement in dual practice in three African capital cities
Source: Health Policy Plan. 2013 Sep 26;29(6):774–83. doi: 10.1093/heapol/czt071 (PMC4153303; doi:10.1093/heapol/czt071)
Supplement: Translated Abstracts [file supp_czt071_czt071_French.pdf]

Secteur de la santé: une étude sur les médecins exerçant la double pratique dans trois capitales africaines

Giuliano Russo,[1]* Barbara McPake,[2] Inês Fronteira [1] and Paulo Ferrinho [1]

[1] International Public Health and Biostatistics Unit, Instituto de Higiene e Medicina Tropical, Universidade Nova de Lisboa and Centre for Malaria and Tropical Diseases, Associated Laboratory, Lisbon, Portugal and [2] Institute for International Health Development, Queen Margaret University, Edinburgh, UK

*Corresponding author. Instituto de Higiene e Medicina Tropical, Universidade Nova de Lisboa, Rua da Junqueira 100, 1349-008 Lisbon, Portugal. Email: grusso@ihmt.unl.pt

Accepté le 23 août 2013

Nous ne savons pas grand-chose sur les caractéristiques, les facteurs déterminants et les implications des médecins exerçant la double pratique, surtout dans un environnement pauvre en ressources. L'étude met à jour cette double pratique dans trois capitales africaines et nous éclaire aussi sur la structure des marchés des services médicaux respectivement proposés dans ces villes avec pour objectif de comprendre les facteurs qui influencent cette pratique. Quarante-huit entretiens qualitatifs approfondis ont été menés dans les trois capitales afin de comprendre les caractéristiques de cette pratique ainsi que celles du marché. Une étude a été réalisée sur un échantillon de 331 médecins afin de comprendre leurs caractéristiques et leurs décisions de travailler dans le secteur public et dans le secteur privé. Des analyses et des statistiques ont été utilisées pour étudier les différences entre les médecins ayant recours à la double pratique dans les trois villes concernées. Différentes formes de double pratique ont été identifiées dans ces villes, avec des médecins du secteur public travaillant pour le privé à l'extérieur mais aussi au sein d'un établissement public de façon réglementée ou non. 34% des personnes ayant participé à l'étude ont indiqué qu'elles travaillaient seulement pour le secteur public, 11% seulement pour le secteur privé. Les 55% restant ont dit avoir recours à la double pratique, 31% à l'extérieur de l'établissement public, 8% à l'intérieur et 16% à l'extérieur et à l'intérieur. La gouvernance locale du système de santé et la structure du marché sont liées aux différentes formes de double pratique et à leur prévalence. Nos analyses suggèrent que les décisions des médecins d'avoir recours à la double pratique sont influencées par des facteurs d'offre et de demande mais aussi par la séparation assez claire entre le secteur public et le secteur privé. Là où il est possible de fournir un service équitable, efficace et un peu réglementé dans des infrastructures publiques, on observe qu'il y a moins de tentations à exercer dans le secteur privé. L'étude montre ce qu'apporte le fait d'analyser le marché de la santé pour comprendre l'engagement des médecins dans des activités professionnelles et contribue à mettre en place une base pour sa réglementation.

Mots clés : Double pratique, emploi multiple, ressources humaines dans la santé, médecins en Afrique, Cap Vert, Guinée Bissau, Mozambique, système de recherche de santé dans des pays à faible revenu
