# Supplementary material for: Negotiating markets for health: an exploration of physicians’ engagement in dual practice in three African capital cities
Source: Health Policy Plan. 2013 Sep 26;29(6):774–83. doi: 10.1093/heapol/czt071 (PMC4153303; doi:10.1093/heapol/czt071)
Supplement: Translated Abstracts [file supp_czt071_czt071_Spanish.pdf]

Negociación de los mercados de la salud: una exploración de los compromisos de los médicos en la práctica dual en tres capitales africanas

Aceptado 23 de Agosto de 2013

Existe escasa evidencia de las características, los determinantes y las implicaciones de la práctica dual de los médicos especialmente en lugares de escasos recursos. Este estudio examina patrones de la práctica dual en tres ciudades de África y los respectivos mercados para servicios médicos, con el objetivo de comprender la influencia de los determinantes locales en la práctica. Se realizaron cuarenta y ocho entrevistas cualitativas semiestructuradas en las tres ciudades para entender las características de la práctica y los mercados respectivos. Se realizó una encuesta en una muestra de 331 médicos para explorar sus características y sus decisiones de trabajar en los sectores público y privado. Se utilizaron análisis descriptivos y estadísticas inferenciales para explorar las diferencias en el compromiso de los médicos en la práctica dual a través de las tres ubicaciones. Se encontraron que existían diferentes formas de la práctica dual en las tres ciudades, con los médicos públicos participando en la práctica privada, afuera pero también dentro de las instalaciones públicas, en formas tanto reguladas como no reguladas. Treinta y cuatro por ciento de los encuestados manifestaron que ellos trabajaban solamente en la práctica pública, y 11% que ellos participaban exclusivamente en la práctica privada. El restante 55% indicó que ellos estaban involucrados en alguna forma de práctica dual, 31% 'fuera' de instalaciones públicas, 8% 'dentro' y 16% en ambas, 'fuera' y 'dentro'.

La gobernanza del sistema de salud local y la estructura de los mercados para los servicios médicos estaban vinculadas a las formas de práctica dual encontradas en cada lugar y a su prevalencia. Nuestro análisis sugiere que las decisiones de los médicos de dedicarse a la práctica dual están influenciadas por factores de oferta y demanda, pero también por que tan claramente separados estén los mercados públicos y privados. Donde es posible suministrar servicios poco regulados dentro de la infraestructura pública, parecen existir menos incentivos para involucrarse en el sector formal privado, con implicaciones de equidad y eficiencia para la prestación del servicio. El estudio muestra el valor de analizar los mercados de la salud para comprender el compromiso de los médicos en las actividades profesionales y contribuye a una evidencia para su regulación.

Palabras claves

práctica dual, pluriempleo, recursos humanos para la salud, médicos en África, Cabo Verde, Guinea Bissau, Mozambique, investigación del sistema de salud en países de bajos ingresos

Mensajes claves

Se encontraron diferentes formas de práctica dual que existen en tres capitales africanas, con médicos públicos involucrados en prácticas privadas afuera pero también adentro de las instalaciones públicas, en formas reguladas como no reguladas.

Treinta y cuatro por ciento de los médicos indicaron que están vinculados solamente con la práctica pública. El 11% indicó que participan en la práctica privada solamente. El restante 55% indicó que ellos ejercen en alguna forma

la práctica dual. La proporción de practicantes duales se encontró consistente en las tres ubicaciones.

La gobernanza del sistema local de salud y la estructura de los mercados para los servicios médicos estaban vinculadas a las formas de práctica dual encontrada en cada lugar y a su prevalencia.

Analizar la estructura y las características de los mercados locales de servicios médicos es clave para entender las decisiones de los médicos de participar en la práctica dual, y para identificar las opciones de políticas adecuadas.
