# Supplementary material for: Negotiating markets for health: an exploration of physicians’ engagement in dual practice in three African capital cities
Source: Health Policy Plan. 2013 Sep 26;29(6):774–83. doi: 10.1093/heapol/czt071 (PMC4153303; doi:10.1093/heapol/czt071)
Supplement: Supplementary Data [file supp_czt071_suppl_data.zip › Revised Annex I_Statistical annex_220713.doc]

# Annex I: Statistical annex

Table S1: People interviewed by location and professional group

| Location | Public sector only physicians | Private sector only physicians | Physicians working both in public and private | Health policy-makers | Total |
| --- | --- | --- | --- | --- | --- |
| Cape Verde | 4 | 1 | 7 | 5 | 17 |
| Guinea Bissau | 1 | 3 | 6 | 6 | 16 |
| Mozambique | 2 | 1 | 7 | 5 | 15 |
| Total | 7 | 5 | 20 | 16 | 48 |

Table S2: Survey population in the three capital cities

|  | Estimated physician population | Physicians accepting to participate | Physicians refusing to participate | Unable to locate |
| --- | --- | --- | --- | --- |
| Praia | 138 | 110 (79.7%) | 8 (5.8%) | 20 (14.5%) |
| Bissau | 117 | 95 (81.2%) | 5 (4.3%) | 17 (14.5%) |
| Maputo | 382 | 125 (32.7%) | 32 (8.4%) | 225 (58.9%) |
| Total | 637 | 330 (51.8%) | 45 (7.1%) | 262 (41.1%) |

Table S3: Descriptive statistics for surveyed physicians

|  | **Praia** | | **Bissau** | | **Maputo** | | **Total** | |
| --- | --- | --- | --- | --- | --- | --- | --- | --- |
| **Physicians surveyed (N)** | 110 | | 95 | | 125 | | 330 | |
| **Sex (n; %)** | | | | | | | | |
| Male | 48 | 43.6 | 69 | 71.9 | 62 | 49.6 | 179 | 54.1 |
| Female | 62 | 56.4 | 27 | 28.1 | 63 | 50.4 | 152 | 45.9 |
| **Years of age – mean (sd)** | 43.6 (10.6) | | 49.3 (6.5) | | 42.3 (9.5) | | 44.8 (9.5) | |
| **Civil status (n; %)** | | | | | | | | |
| Married | 70 | 63.6 | 76 | 80.0 | 96 | 76.8 | 242 | 73.1 |
| Single | 40 | 36.4 | 19 | 20.0 | 29 | 23.2 | 88 | 26.7 |
| **Dependents (n; %)** | | | | | | | | |
| No | 17 | 15.5 | 6 | 6.3 | 7 | 5.6 | 30 | 9.1 |
| Yes | 93 | 84.5 | 90 | 93.8 | 118 | 94.4 | 301 | 90.9 |
| **N of dependents – mean (sd)** | 2.0 (1.1) | | 7.7 (4.0) | | 2.8 (1.6) | | 4.1 (3.5) | |
| **Doctor in the family (n; %)** | | | | | | | | |
| No | 45 | 41.3 | 56 | 58.9 | 74 | 59.7 | 175 | 53.4 |
| Yes | 64 | 58.7 | 39 | 41.1 | 50 | 40.3 | 153 | 46.6 |
| **Years of practice – mean (sd)** | 11.6 (8.9) | | 12.8 (7.4) | | 10.2 (8.4) | | 11.4 (8.3) | |
| **Practice outside the capital city (n; %)** | | | | | | | | |
| No | 88 | 80.7 | 85 | 88.5 | 101 | 80.8 | 274 | 83.0 |
| Yes | 21 | 19.3 | 11 | 11.5 | 24 | 19.2 | 56 | 17.0 |
| **Specialty (n; %)** | | | | | | | | |
| No | 38 | 34.9 | 22 | 22.9 | 55 | 44.0 | 115 | 34.8 |
| Yes | 71 | 65.1 | 74 | 77.1 | 70 | 56.0 | 215 | 65.2 |
