# Supplementary material for: Negotiating markets for health: an exploration of physicians’ engagement in dual practice in three African capital cities
Source: Health Policy Plan. 2013 Sep 26;29(6):774–83. doi: 10.1093/heapol/czt071 (PMC4153303; doi:10.1093/heapol/czt071)
Supplement: Supplementary Data [file supp_czt071_suppl_data.zip › Annex II_Interview guide.doc]

# Annex II: Dual practice interview guide

## Introduction

Good morning, I am a researcher from the IHMT, and I am conducting a study sponsored by the WHO and Gulbenkian Foundation on the engagement of public sector physicians in private sector activities.

All the information you are willing to facilitate will be treated confidentially, and your name will not appear anywhere in the report – read out the confidenciality form

## General information on dual practice (understanding dual practice in the country)

1. Where do you work and what is you area of specialisation?
2. Are you familiar with physicians’ practice of engaging in the public and private at the same time?
3. How does it work in your country, what medical doctors do, in practice?
   a. Probe: work in public in the morning, private in afternoon
   b. Probe: formal Contracts with public sector.
4. Is such practice popular in your country, and in what geographical parts of the country is more common?
5. In what services and specialisations is most common to see physician DP?
   a. Probe: (inpatient/outpatient/other) – (specialty)
6. How much does a doctor make per month in the public sector?
7. How many hours per week does she typically work?
8. What is the typical fee charged for a private visit?

## Incentives

9. Do you practice in the public as well as in the private sector?
10. How did you get engaged with the practice?
    a. Probe: money, opportunity, peers pressure
11. How many hours per week do you work in the public and how many in
12. What are the main benefits of working for the private?
13. Why do you (and your colleagues) still work in the public sector?
    a. Probe: credibility, stability, hope for improvement in public services, humanitarian reasons, need to be there to access private customers)
14. What would happen if you didn't turn up at the public job at all? Only turned up for 2 hours/day... (these need to be referenced to an understanding of existing norms).
    a. Probe: disciplinary action (make sure you explore the likely consequences of that if it's raised);
    b. Probe: loss of private patients (then you need to explore how private patients are generated by public practice eg. whether this is a way of generating initial consultations that are then followed by a flow of further consultations; or whether most consultations will start in the public facility and be referred loss of job and security??
15. Has this changed (explore been growing; reducing; why –… before getting into regulation).
16. How many private patients do you see in a week?

## Impact of regulation

17. Can you recall any specific event or circumstance in your professional career that pushed you towards or away from the private sector?
18. Are you aware of any attempt by the Government to regulate DP? What is the Government doing/did in the past to regulate the practice?
    a. Probe: Explore any discrete changes in the past
19. Do you think it was successful? And if not, why was it not?
20. In what respect was the situation different before the attempt of regulation?
21. In your opinion, what are the pros and cons of the practice?
22. What do you think the Government should do to improve the practice?
    a. Probe: what sort of incentives would you need to dedicate more time to the public sector?

Many thanks for your help. Is there any other relevant piece of information you would like to share with us on dual practice that we forgot to as
